# The thrombotic paradox in congenital fibrinogen deficiencies: from pathophysiology to practice

**DOI:** 10.1016/j.rpth.2025.102979

**Published:** 2025-07-22

**Authors:** Samin Mohsenian, Alessandro Casini, Flora Peyvandi

**Affiliations:** 1 Department of Pathophysiology and Transplantation, Università degli Studi di Milano, Milan, Italy; 2 Division of Angiology and Hemostasis, Faculty of Medicine, Geneva University Hospitals, Geneva, Switzerland; 3 Fondazione IRCCS Ca’Granda Ospedale Maggiore Policlinico, Angelo Bianchi Bonomi Hemophilia and Thrombosis Center and Fondazione Luigi Villa, Milan, Italy

**Keywords:** afibrinogenemia, blood coagulation disorders, congenital fibrinogen deficiencies, dysfibrinogenemia, fibrinogen, hypofibrinogenemia, hypodysfibrinogenemia, management, risk factors, thrombophilia, venous thrombosis

## Abstract

Congenital fibrinogen deficiencies (CFDs) comprise rare inherited disorders characterized by quantitative (afibrinogenemia, hypofibrinogenemia) or qualitative (dysfibrinogenemia, hypodysfibrinogenemia) abnormalities of fibrinogen. While CFDs are typically associated with bleeding, a paradoxical risk of both arterial and venous thrombosis is being increasingly recognized. Proposed mechanisms include impaired thrombin clearance due to a lack of fibrin formation and structurally abnormal fibrin clots that promote thrombin release into the circulation or hinder fibrinolysis. In afibrinogenemia, the absence of fibrinogen leads to increased circulating free thrombin, while in dysfibrinogenemia, structurally abnormal fibrinogen enhances thrombotic risk. Intrinsic factors such as specific fibrinogen variants (eg, Dusart, Bordeaux) alter the fibrin structure and impair thrombin or plasmin interactions, thus promoting abnormal clot formation and reduced fibrinolysis. Coinherited prothrombotic mutations may further increase thrombotic risk. Moreover, acquired factors, including fibrinogen replacement therapy, surgery, trauma, pregnancy, and immobilization, are recognized extrinsic risk factors. The pathogenesis of thrombosis in CFDs is multifactorial and not fully elucidated. Managing thrombosis in CFDs is a clinical challenge, requiring careful balance between the risk of bleeding and thrombosis. Anticoagulation alongside fibrinogen replacement may be necessary, but must be individualized. Although fibrinogen replacement primarily carries prothrombotic potential, some studies suggest it may improve thrombin regulation in afibrinogenemia. Notably, current evidence is limited and mostly derived from case reports. This review provides an overview of the existing evidence on the epidemiology, underlying mechanisms, and clinical management of thrombosis in CFDs, highlighting knowledge gaps and the need for additional research to inform clinicians and improve patient outcomes.

## Essentials

• Although CFDs involve bleeding, thrombosis also occurs.
• This review comprehensively summarizes current understanding of thrombosis in CFDs.
• Genetic mutations and extrinsic factors might affect thrombosis risk in CFDs.
• Managing thrombosis in CFDs requires balancing bleeding risk and clotting tendency.

## 1 Introduction

Fibrinogen, also known as coagulation factor I, is a 340-kDa glycoprotein complex produced in the liver and circulating in blood [1]. It consists of 3 pairs of polypeptide chains (Aα, Bβ, and γ), encoded by 3 different genes, *FGA*, *FGB*, and *FGG*, respectively [2]. As the precursor of fibrin, this plasma protein is converted by thrombin during coagulation to form a stabilizing fibrin mesh that prevents excessive bleeding and supports wound healing [3]. Congenital fibrinogen deficiencies (CFDs) are a group of rare inherited disorders, classified as either quantitative (type 1) or qualitative (type 2) deficiencies based on plasma fibrinogen levels [4]. Quantitative deficiencies include afibrinogenemia, defined by a nearly complete absence of fibrinogen, and hypofibrinogenemia, defined by reduced fibrinogen activity and antigen levels. Qualitative deficiencies include dysfibrinogenemia, with reduced fibrinogen activity but normal antigen levels, and hypodysfibrinogenemia, with a disproportionate decrease in both activity and antigen [4]. Given that fibrinogen is also essential for placental maintenance, early trophoblast proliferation, and migration, as well as the establishment of the maternal–fetal circulation, women with CFD face an increased risk of adverse obstetric outcomes [5,6].

While CFDs are traditionally associated with a bleeding tendency due to impaired clot formation, a paradoxical predisposition to thrombosis has been increasingly reported, potentially affecting both arterial and venous vessels [[7], [8], [9], [10], [11], [12]]. According to the International Society on Thrombosis and Haemostasis classification for CFDs, afibrinogenemia 1B is defined by a thrombotic phenotype affecting venous and/or arterial territories. Similarly, type 3B dysfibrinogenemia is characterized by the presence of a prothrombotic fibrinogen mutation or a history of thrombotic events in individuals with a first-degree relative affected by thrombosis, in the absence of other thrombophilic conditions [13]. The overall rate of thrombosis in CFDs has been reported at approximately 20%, highlighting the paradoxical thrombotic tendency observed in these conditions [14]. This dual hemostatic imbalance poses a major clinical challenge for both diagnosis and management. The mechanisms underlying the thrombotic risk in CFDs remain poorly understood but are thought to involve impaired thrombin binding and sequestration, alterations in fibrin clot structure, compensatory hypercoagulability, and genetic factors [15]. The dual risks of bleeding and thrombosis in CFDs often necessitate the simultaneous use of anticoagulants and fibrinogen replacement to balance these opposing risks [14,16]. Given the rarity of these disorders, data on thrombotic risk and optimal management strategies are limited, often relying on case reports and small cohort studies.

With this background, this review aims to provide a comprehensive overview of thrombosis in CFDs, summarizing the current understanding of its frequency, pathophysiology, risk factors, clinical manifestations, and management strategies. By integrating recent findings and clinical experiences, we seek to enhance awareness of this underrecognized complication, address future research, and approach patient care.

## 2 Methods

A comprehensive literature review was conducted to identify studies reporting thrombotic events in patients with CFDs. Relevant publications were retrieved through structured searches of databases such as PubMed and Scopus using keywords including “congenital fibrinogen deficiency,” “afibrinogenemia,” “hypofibrinogenemia,” “dysfibrinogenemia,” “hypodysfibrinogenemia,” “thrombosis,” “arterial,” “venous,” “pathophysiology,” “risk factors,” and “management.”

Studies were included if they reported the number or percentage of thrombotic events and specified the type of thrombosis (arterial or venous) in clearly defined patient populations. Data were extracted on the number of patients with CFD, the subtype of deficiency, and the occurrence and type of thrombosis.

Chi-squared tests were used to compare the distribution of thrombotic events across CFD subtypes. Specifically, comparisons were made between (1) the overall rate of thrombosis and (2) the distribution of venous versus arterial thrombosis across different CFD subtypes. *P* < .05 was considered statistically significant. All statistical analyses were performed using R-Studio (2022.7.1 “Foundation” release) as the integrated development environment for all R-based computations.

## 3 Frequency and type of Thrombosis

Individual cases of patients with thrombosis have been reported in systematic literature reviews [15,17,18]. The likelihood of thrombosis can vary depending on individual patient characteristics, specific genetic mutations, and study methodologies. Additional risk factors, such as surgery, pregnancy, or other prothrombotic conditions, may further contribute to the incidence of thrombotic events. Several studies have detailed thrombotic outcomes in large series of patients with prevalences varying from 4% to 44% depending on the type of CFD (Table 1) [7,8,12,[19], [20], [21], [22], [23], [24], [25], [26], [27], [28], [29], [30], [31], [32], [33], [34], [35], [36], [37], [38], [39], [40], [41], [42]]. This variability in thrombosis rates underscores the complex and multifactorial nature of CFD pathophysiology.

**Table 1:** Case series of patients with congenital fibrinogen disorders reporting on thrombotic events.

Authors	Study population	Overall patients with thrombosis, *n* (%)	Venous, *n* (%)	Arterial, *n* (%)	Type of thrombosis
Nguyen et al., 1998 [19]	Dysf, *n* = 12	4 (33.3)	4 (33.3)	0 (0)	4 DVT, 1 with PE
Lak et al., 1999 [20]	Afib, *n* = 55	2 (4.0)	1 (1.8)	1 (1.8)	1 CVT and 1 ALI
Kreuz et al., 2005 [21]	Afib, *n* = 12 Hypo, *n* = 3 Dysf, *n* = 1	1 (6.3)	1 (6.3)	0 (0)	1 PE and 1 DVT in a patient with Afib
Santacroce et al., 2006 [22]	Afib, *n* = 4 Hypo, *n* = 5 Dysf, *n* = 9	1 (5.5)	1 (5.5)	1 (5.5)	1 CVT in Dysf
Miesbach et al., 2010 [23]	Dysf, *n* = 37	9 (24.3)^a^	2 (5.4)	7 (18.9)	2 DVT; 3 strokes, 3 ALI, 1 AMI, 1 retinal artery thrombosis
Shapiro et al., 2013 [24]	Dysf, *n* = 35	3 (9)	2 (5.7)	1 (2.8)	2 PE; 1 stroke
Casini et al., 2015 [25]	Dysf, *n* = 101	28 (27.7)^b^	20 (19.8)	8 (7.9)	11 DVT, 3 PE, 2 SVT, 3 PT, 1 RVT; 4 strokes, 2 AMI, 1 ALI, 1 mesenteric thrombosis
Zhou et al., 2015 [26]	Dysf, *n* = 102	4 (3.9)	4 (3.9)	0 (0)	2 DVT, 1 PE, 1 PT
Asselta et al., 2015 [27]	Dysf, *n* = 7	1 (14.2)	1 (14.2)	0 (0)	1 CVT
Asselta et al., 2015 [28]	Afib, *n* = 13 Hypo, *n* = 8	2 (9.5)	2 (9.5)	0 (0)	2 DVT in Hypo
Mukaddam et al., 2015 [29]	Afib, *n* = 13 Dysf, *n* = 3	1 (6.25)	1 (6.25)	0 (0)	1 PT in Afib
Casini et al., 2015 [30]	Dysf, *n* = 24	8 (33.3)^c^	7 (29.1)	1 (4.1)	3 DVT, 1 PE, 1 PT, 1 CVT, 1 RVT; 1 stroke
Rottenstreich et al., 2015 [8]	Afib, *n* = 5 Hypo, *n* = 4	4 (44.4)^d^	3 (33.3)	1 (11.1)	1 PVT, 1 adrenal vein thrombosis, 1 DVT; 1 AMI all in Afib
Négrier et al., 2016 [31]	Afib, *n* = 14	1 (7.1)	1 (7.1)	0 (0)	1 DVT
Chinni et al., 2018 [32]	Afib, *n* = 1 Hypo, *n* = 6 Dysf, *n* = 3	2 (20)	2 (20)	NA	1 CVT in Afib, 1 DVT in Dysf
Smith et al., 2018 [33]	Afib, *n* = 3 Hypo, *n* = 11 H/Dysf, *n* = 17	6 (19.3)	4 (12.9)	2 (6.4)	1 PE, 1 stroke in Afib; 1 AMI, 1 PE, 2 DVT in H/Dysf
Castaman et al., 2019 [34]	H/Dysf, *n* = 50	4 (8)	2 (4)	2 (4)	2 DVT; 2 strokes
Djambas Khayat et al., 2019 [35]	Afib, *n* = 14	2 (14.2)	2 (14.2)	0 (0)	2 SVT^e^
Wypasek et al., 2019 [36]	Afib, *n* = 1 Hypo, *n* = 12 H/Dysf, *n* = 13	3 (11.1)	3 (11.1)	0 (0)	1 DVT in H/Dysf, 1 SVT in Dysf, 1 CVT in Dysf
Lasky et al., 2020 [37]	Afib, *n* = 13 Hypo, *n* = 6 H/Dysf, *n* = 3	2 (9.1)	2 (9.1)	0 (0)	1 PE in Afib; 1 SVT in H/Dysf
Zhou et al., 2021 [38]	Hypo, *n* = 2 H/Dysf, *n* = 19	1 (4.7)	0 (0)	1 (4.7)	1 AMI in Dysf
Casini et al., 2021 [7]	Afib, *n* = 204	37 (18.1)	18 (8.8)	19 (9.3)	8 DVT, 5 PT, 3 PE, 1 renal vein thrombosis, 1 CVT, 7 ALI, 6 strokes, 2 AMI, 2 splenic thrombosis, 1 aortic thrombosis, 1 renal artery thrombosis
Hadjali-Saichi et al., 2021 [39]	Afib, *n* = 46	7 (15.2)	3 (6.5)	4 (8.6)	1 DVT, 1 PE with DVT, 1 PT; 4 strokes
Mohsenian et al., 2023 [12]	Afib, *n* = 30 Hypo, *n* = 33 Dysf, *n* =55 H/Dysf, *n* = 5	9 (7.3)	NA	NA	3 Afib, 3 Hypo, 3 Dysf
Hugon-Rodin et al., 2023 [40]	Hypo, *n* = 49 H/Dysf, *n* = 110	21 (13.4)	18 (11.3)	3 (1.8)	9 VT in Hypo, 9 VT in H/Dysf; 2 AT in Hypo, 1 AT in H/Dysf
Cai et al., 2025 [41]	Afib, *n* = 1 Hypo, *n* = 8 H/Dysf, *n* = 42	8 (15.7)	8 (15.7)	0 (0)	1 CVT in Afib; 1 PE in Hypo; 2 PE, 1 DVT, 3 CVT in H/Dysf
Haisma et al., 2025 [42]	Severe Hypo/Afib: 14 Moderate Hypo: 11 Mild Hypo: 6 Carriers without Hypo: 6 H/Dysf: 10	10 (21.2)	6 (13)	4 (9)	3 DVT in Severe Hypo/Afib, 2 DVT in Moderate Hypo, 1 DVT in Dysf. 2 AT in Hypo and 2 AT in H/Dysf. All AT were associated with acute coronary syndromes or strokes

Due to the limited number of patients with hypodysfibrinogenemia, the data were recorded as dysfibrinogenemia.

Afib, afibrinogenemia; ALI, acute limb ischemia; AMI, acute myocardial infarction; AT, arterial thrombosis.; CVT, cerebral veins thrombosis; DVT, deep venous thrombosis; Dysf, dysfibrinogenemia; H/Dysf, hypofibrinogenemia/dysfibrinogenemia; Hypo, hypofibrinogenemia; NA, not available; PE, pulmonary embolism; PT, portal thrombosis; RVT, retinal vein thrombosis; SVT, superficial venous thrombosis; VT, venous thrombosis.

a First thrombotic events. Two patients had recurrent arterial events.

b First thrombotic events. Eight patients had recurrent arterial (*n* = 3) or venous (*n* = 5) events.

c First thrombotic event. Three patients had recurrent venous events.

d First thrombotic event. Two patients had recurrent arterial (*n* = 1) or venous (*n* = 3) events.

e Asymptomatic SVT, diagnosed by systematic venous compression ultrasonography.

In afibrinogenemia, reported thrombosis rates range from 4% to 44% [8,20,31], whereas in dysfibrinogenemia, the prevalence similarly varies between 4% and 33% (Table 1) [23,30,40]. In 2 cohorts of 33 and 49 patients with hypofibrinogenemia, the prevalence of thrombosis was 10% and 22%, respectively [12,23]. Nevertheless, when study populations were pooled, no statistically significant difference (*P* = 0.807) in overall thrombosis rates was observed among the different subtypes of CFDs (Table 2) [7,8,12,[20], [21], [22], [23], [24], [25], [26], [27], [28], [29], [30], [31], [32], [33], [34], [35], [36], [37], [38], [39], [40], [41], [42], [43], [44]].

**Table 2:** Summary of reported thrombotic events in patients with congenital fibrinogen deficiencies.

Type of fibrinogen disorder	Total thrombosis, *n* (%)	Venous thrombosis^a^, *n* (%)	Arterial thrombosis^a^, *n* (%)	References
Afibrinogenemia (*n* = 443)	66 (15)	37 (59)	26 (41)	[7,8,12,[20], [21], [22],[28], [29], [30], [31],^33^,[35], [36], [37],^39^,^41^,^42^]
Hypofibrinogenemia (*n* = 164)	21 (13)	14 (78)	4 (22)	[8,12,21,22,28,30,33,36,38,[40], [41], [42], [43]]
Hypo/dysfibrinogenemia (*n* = 658)	94 (14)	67 (74)	24 (26)	[12,19,21,30,[32], [33], [34],^36^,^37^,^39^,^40^,^42^,^44^]

a Percentages of venous and arterial thrombosis are based only on studies that specifically reported the type of thrombotic events.

Of note, this pooled analysis has some limitations. Data are based on published cases, likely favoring unusual or severe events. Diagnostic criteria varied, and asymptomatic thrombosis may be underdetected. Additionally, some thrombotic events may have occurred in undiagnosed CFD patients. Therefore, larger population-based studies and future systematic studies are needed to clarify thrombosis patterns in CFD.

Overall, thrombotic manifestations covered a broad spectrum, from traditional venous territories to such atypical presentations as splanchnic, renal, spinal or cerebral veins. A high prevalence of aortic thrombi and digital ischemia was observed, especially in cases of afibrinogenemia [18,20,23,30,31].

In our previous report from the Prospective Rare Bleeding Disorders Database (PRO-RBDD) study, which investigated 123 cases of CFDs, 56% of thrombosis events occurred in venous vessels, 22% in arterial, and 22% in both territories. Notably, the occurrence of thrombosis in both territories was observed exclusively in cases of afibrinogenemia and hypofibrinogenemia [12].

In a large cohort of cases with afibrinogenemia (*n* = 204), 37 (18.1%) of them experienced a thrombotic event, with 16 (43.3%), 11 (29.7%), and 10 (27%) reporting events in venous, arterial, or both territories [7]. Venous thromboses occurred in young patients with a mean age at first event of 27 years, including 6 children (7%), while arterial thromboses were observed only in adults, with a mean age of 36 years at the first event. Of note, all thrombotic events (7%) in children (aged 4-15 years) were venous, while in adults, the events were almost equally distributed between the venous (32%) and arterial (35%) territories [7]. Thrombotic recurrence occurred in 15 (40.5%) patients, in both venous (*n* = 6) and arterial (*n* = 9) vessels. Four patients experienced multiple recurrences of acute peripheral ischemia of toes [7].

Historically, it has been reported that 55% of patients with dysfibrinogenemia are asymptomatic, 25% exhibit a tendency to bleeding, and 20% have a thrombotic tendency [45]. However, the most recent classification of dysfibrinogenemia has highlighted the higher thrombotic risk conferred by type 3B dysfibrinogenemia [13]. A seminal report by Haverkate and Samama [46] in 1995 observed that all cases of thrombotic-related dysfibrinogenemia (ie, type 3B) and the majority of carrier relatives exhibited venous and/or arterial thrombosis since early adulthood. More recently, fibrinogen Paris V, clinically referred to as Dusart syndrome, was observed in a dysfibrinogenemic Scandinavian family, characterized by the early onset of arterial and venous thrombosis [47].

An international prospective study investigated the prevalence of thrombosis in dysfibrinogenemia, including a cohort of 101 cases with dysfibrinogenemia [25]. At the time of diagnosis, 13.9% of cases had experienced a thrombotic event. During a mean follow-up of 8.8 years after diagnosis, the incidence of thrombotic events was 7.6 per 1000 patient-years, with an estimated cumulative incidence at 50 years of 30.1% (95% CI, 20.1%-43.5%). Although venous thrombosis events were more frequent than arterial (5.7 vs 2.6 per 1,000 patient-years), the distribution of thrombosis types did not differ significantly across CFD subtypes (*P* = 0.09; Table 2). In the context of age- and sex- adjusted Cox regression analyses, no statistically significant difference in the overall risk of thrombotic events was observed between women and men (hazard ratio, 0.9; 95% CI, 0.4-1.9) [25]. At variance with these findings, other studies reported a lower prevalence of thrombosis (Table 1). However, notwithstanding the aforementioned study, most published cohorts of patients reported only thrombotic events at diagnosis without follow-up.

## 4 Pathophysiology of Thrombosis in CFD

### 4.1 Impaired thrombin binding and sequestering

In afibrinogenemia, the main mechanistic explanation of thrombosis is the complete absence of fibrinogen, which prevents thrombin from being sequestered in fibrin clots, impairs its clearance, and leads to high circulation levels of free thrombin that promote excessive clot formation and increase thrombotic risk [48]. Although residual fibrinogen in hypofibrinogenemia has been hypothesized to regulate thrombin generation and clot formation, clinical data indicate that thrombosis can occur in both afibrinogenemia and hypofibrinogenemia, with comparable incidence rates [9]. Notably, low fibrinogen levels do not appear to offset a hypercoagulable state [49]. The exact mechanisms underlying thrombosis in these conditions remain not fully understood.

The second potential mechanism may involve increased levels of prothrombin activation fragments or thrombin–antithrombin complexes, which indicate enhanced thrombin generation [50]. Supporting studies have demonstrated increased thrombin generation in these contexts [51,52], and recent work has linked this enhanced thrombin activity to a higher risk of thrombosis [42].

Another possible explanation is that thrombin activates platelets, leading to the release of growth factors that drive smooth muscle proliferation and intimal hyperplasia. In afibrinogenemia, recurrent bleeding may result in local thrombin generation; in the absence of a stabilizing fibrin cap, unbound thrombin may further promote platelet activation and smooth muscle migration. This sequence of events could increase intimal vulnerability and the risk of embolization, making afibrinogenemia a paradoxical vascular risk factor [53].

### 4.2 Abnormal fibrin clot structure

In qualitative fibrinogen deficiencies, 2 general mechanisms have been proposed [54]. The primary mechanism is impaired anticoagulant function, caused by variants that disrupt thrombin binding to low-affinity sites on fibrin. This defect likely leads to the release of free thrombin into circulation, which promotes excessive fibrin clot formation and heightens the risk of thrombosis [54].

The second mechanism involves impaired fibrinolysis due to variants that disrupt the binding of such profibrinolytic proteins as tissue plasminogen activator and plasminogen to fibrin. Additionally, these variants may increase fibrin’s resistance to plasmin-mediated degradation. Together, these defects contribute to a heightened risk of thrombosis.

### 4.3 Platelet aggregation via von Willebrand factor

Another possible explanation is that, even in the absence of fibrinogen, von Willebrand factor (VWF) may interact with the platelet integrin glycoprotein IIb/IIIa and could potentially serve as an alternative mediator of platelet aggregation [55]. However, there is currently no direct evidence that VWF-mediated aggregation promotes a prothrombotic state in afibrinogenemia, and its overall contribution to thrombosis remains unclear. In the absence of fibrin, these aggregates are fragile and prone to disaggregation under shear stress, potentially favoring embolization rather than stable thrombus formation. This interpretation is supported by findings from a zebrafish model [56] in which VWF-mediated platelet aggregation failed to produce sustained thrombi but was associated with embolic phenomena. Thus, the role of VWF in the thrombotic complications of CFD is likely indirect, context-dependent, and part of a multifactorial process.

## 5 Thrombosis Risk Factors

### 5.1 Intrinsic: genetic mutations

**Figure 1:**
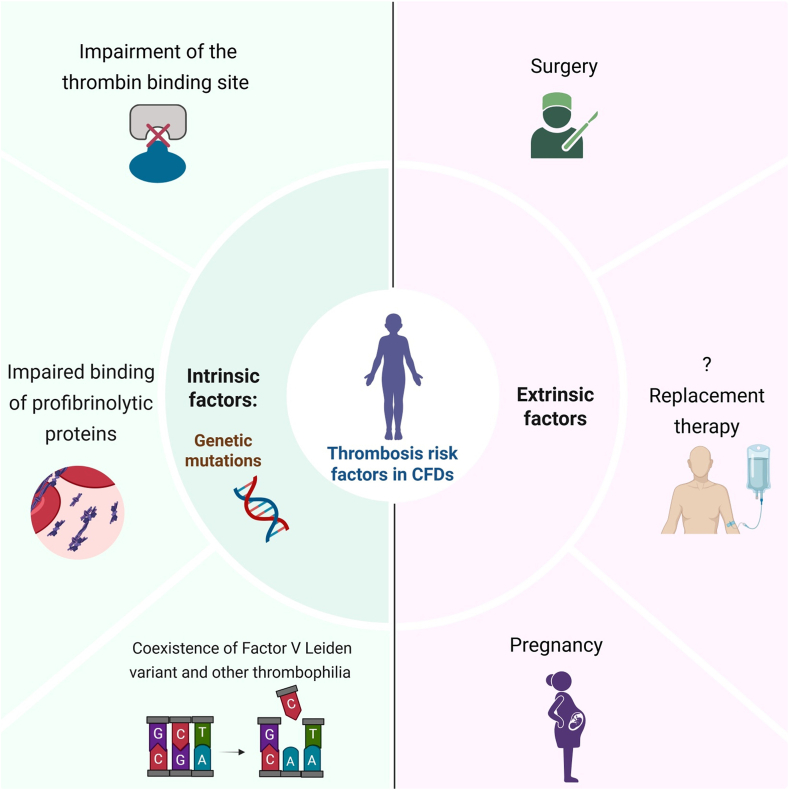
The risk factors associated with thrombosis in congenital fibrinogen deficiencies (CFDs). Both intrinsic and extrinsic factors contribute to an increased risk of thrombosis in CFDs. The question mark (?) indicates limited evidence supporting a direct link between standard administration of fibrinogen concentrate and increased thrombotic risk. Figure created with BioRender.com.

Genetic mutations in dysfibrinogenemia can impair the thrombin binding site on fibrinogen. In addition to thrombin binding, genetic mutations in CFDs can also impair fibrin binding of profibrinolytic proteins, such as tissue plasminogen activator and plasminogen. The genetic mutations responsible for these 2 mechanisms can lead to an increased risk of thrombosis (Figure 1).

**Figure 2:**
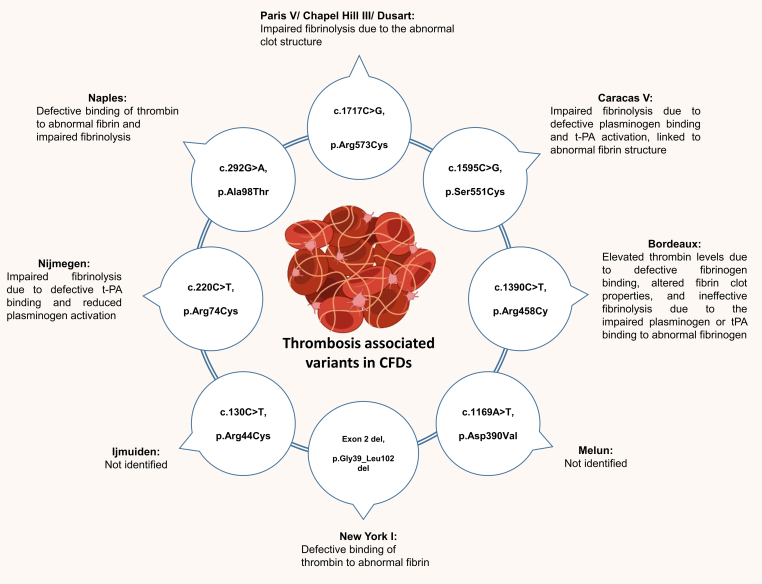
Thrombosis-associated variants in congenital fibrinogen deficiencies. Published variants and their associated pathophysiologic effects on thrombosis. CFD, congenital fibrinogen deficiency; t-PA, tissue plasminogen activator. Figure created with BioRender.com.

The fibrinogen Dusart variant (*FGA*, p.Arg554Cys) is one of the most well-characterized mutations linked to an increased thrombotic tendency in dysfibrinogenemia. Reported cases have shown a high frequency of thromboembolic events and abnormalities in fibrin polymerization. This specific genetic variant appears to compromise clot stability, increasing the likelihood of fragmentation and embolization [46,57]. Similarly, the fibrinogen Bordeaux variant (*FGA,* p.Arg458Cys) leads to impaired and delayed fibrinolysis, associated with thrombosis but without bleeding [58]. The *FGB*, p.Arg44Cys gene variant (fibrinogen Ijmuiden) is also associated with an increased thrombotic tendency in dysfibrinogenemia. This mutation impairs clot formation induced in fibrinogen by thrombin or reptilase, suggesting a defect in fibrin polymerization [59]. The classification of variants such as fibrinogen Baltimore and Bonn as thrombosis-associated dysfibrinogenemias remains inconclusive due to limited and inconsistent clinical and functional evidence [10,59]. Additionally, the association between polymerization defects and thrombosis is not fully understood. While impaired fibrin polymerization is typically associated with bleeding, certain structural abnormalities may lead to the formation of dense, fibrinolysis-resistant clots or altered thrombin interactions, contributing to a prothrombotic state. These complexities underscore the need for more refined criteria to assess the thrombotic risk in dysfibrinogenemia [10,60,61]. The thrombosis-related fibrinogen variants are shown in Figure 2 [46,58,[62], [63], [64], [65], [66], [67], [68]].

Notably, the evaluation of 101 dysfibrinogenemia cases found no significant correlation between fibrinogen activity levels and thrombotic risk, highlighting the role of qualitative defects in the thrombotic predisposition [25].

Several reports have documented thrombotic events in patients with mild to moderate fibrinogen deficiency, especially when accompanied by additional genetic mutations such as the factor (F)V Leiden variant, protein C deficiency, and antithrombin deficiency. The coexistence of CFDs with thrombophilic variants such as the FV Leiden variant presents unique challenges in patient management, as both conditions independently increase the risk of thrombosis (Figure 1). Although there are insufficient studies investigating the coexistence of FV Leiden variant (p.Arg506Gln) and CFDs, a reported case of heterozygous dysfibrinogenemia (*FGG*, p.Arg275Cys) carrying the FV Leiden variant suggests that this combination may increase the risk of thromboembolic events, notwithstanding that most cases with the p.Arg275Cys variant do not experience clinical thrombotic disorders [69]. Another case report described a patient with hypofibrinogenemia who was found to be heterozygous for FV Leiden, indicating that moderate congenital hypofibrinogenemia may not necessarily mitigate the thrombotic risk associated with FV Leiden. This finding supports the consideration of antithrombotic prophylaxis during high-risk situations in patients with this coinheritance; however, given the limited data, such decisions should be made with careful clinical judgment and individualized risk assessment [70].

Combination of protein C deficiency and hypodysfibrinogenemia may predispose to recurrent venous thrombosis [71]. In addition, combined afibrinogenemia and congenital antithrombin deficiency can result in a range of severe manifestations, including spontaneous arterial events such as acute coronary syndrome and stroke; venous thrombosis-like pulmonary embolism; and recurrent, life-threatening bleeding [72].

### 5.2 Extrinsic: *surgery*, *replacement therapy*, and *pregnancy*

In addition to the aforementioned intrinsic risk factors, patients with CFDs are particularly vulnerable to thrombosis in 3 distinct extrinsic clinical settings (Figure 1) [18].

In the context of surgical procedures, the utilization of peripherally inserted central catheters and port-catheters has been associated with an elevated risk of thrombotic events in afibrinogenemia [73,74]. A substantial body of literature documents cases of thrombosis occurring after surgical procedures [75,76]. In a systematic review by Bornikova et al. [18], thrombotic complications were reported in all of the 6 adults with afibrinogenemia undergoing surgery. In these patients, increased fibrinogen supplementation has often been regarded as the initiating factor leading to a hypercoagulable state, although a definitive temporal relationship is not established [12,18]. Poor implementation of thromboprophylaxis due to concerns about bleeding is another potential explanation.

Fibrinogen replacement has frequently been indicated as potentially associated with an increased risk of thrombosis [29,77,78]. This risk should be considered especially when cryoprecipitate is employed as a fibrinogen source, given its content of composition of numerous coagulation proteins in addition to fibrinogen [79]. Thrombotic complications have been reported in clinical studies evaluating the efficacy and safety of fibrinogen concentrates. Two events were assessed as treatment-related: one case involved a total vein thrombosis following splenectomy, considered possibly related to fibrinogen concentrate infusion. In another case, a 32-year-old man with afibrinogenemia developed an asymptomatic distal venous thrombosis of the tibioperoneal vein, which occurred 21 days after his eighth infusion [35,80]. These findings have indeed been confirmed by a prospective multicenter study including patients on fibrinogen concentrate prophylaxis over a median period of 227 days: one pulmonary embolism was documented, occurring 8 days after the last fibrinogen dose [37]. A postauthorization safety study similarly reported only one case of subclavian vein thrombosis in a 35-year-old woman who had received 9 g of fibrinogen in 3 infusions over 4 days [31]. Notably, recent data highlighted differential effects of commercially available fibrinogen concentrates on fibrin clot properties and fibrinolysis [53,81], but further evaluations are necessary to determine the clinical relevance of these changes and their potential impact on the thrombotic risk associated with fibrinogen infusions.

Conversely, fibrinogen concentrates infusion at standard doses has been proposed to reduce the thrombotic risk by lowering the circulating thrombin–antithrombin complex levels. This has been supported by studies showing favorable changes in these levels after fibrinogen administration in cases of afibrinogenemia [17,82]. In qualitative fibrinogen disorders, infusion of normal fibrinogen may dilute abnormal fibrinogen molecules, reducing their likelihood of promoting clot formation. Clots formed in the presence of normal fibrinogen tend to have a more regular structure and are more susceptible to plasmin-mediated degradation [83]. However, it is important to acknowledge that, despite these encouraging findings, comprehensive prospective and long-term studies assessing the safety and efficacy of the currently available fibrinogen concentrates remain limited.

Women with CFDs have an increased risk of obstetrical complications, including thrombosis during pregnancy and the postpartum period [8,37]. This risk has been confirmed by the findings of the Fibrinogest Study, a multicenter, international and prospective study in which 4 of 425 (1%) pregnancies were complicated by thrombotic events in the first trimester, and 5 of 316 (1.6%) deliveries were associated with venous thrombosis in the postpartum period [40]. In type 3B dysfibrinogenemia, the prevalence of thrombosis is particularly prominent in pregnant women. In the aforementioned report by Haverkate and Samama [46], 7 of 15 (47%) of the women had thrombosis in the postpartum period and 1 (7%) during pregnancy. In this setting, additional thrombotic risk factors may contribute to the procoagulant state [84].

## 6 Management of Thrombotic Risk and Its Challenges

The management of thrombotic events in patients with CFDs poses significant challenges to physicians, as they must address both the procoagulant state following acute thrombosis and the risk of bleeding associated with antithrombotic therapy [48,85]. An accurate evaluation of the balance between the bleeding tendency, considering both personal and familial histories, and thrombotic risk is thus critical [86].

The underlying type and subtype of fibrinogen disorder must be considered, as most asymptomatic patients with dysfibrinogenemia or mild hypofibrinogenemia may receive antithrombotic treatment with no need to replace fibrinogen [10,70]. On the other hand, patients with moderate to severe hypofibrinogenemia and afibrinogenemia require fibrinogen replacement to decrease the bleeding risk. While fibrinogen replacement is primarily administered to prevent bleeding, it may also help reduce thrombin–antithrombin complex levels in afibrinogenemia, which might reflect some effect on thrombin regulation, even in cases in which thrombotic events have occurred [17,87].

Although there is a general consensus that fibrinogen levels > 1 g/L may be sufficient for effective hemostasis during antithrombotic treatment, this threshold has yet to be definitively established in patients with quantitative fibrinogen disorders [88].

Treatment decisions must be individualized. While shorter anticoagulation may be appropriate in selected low-risk cases, an extended or indefinite duration should be considered in patients with high thrombotic risk, in accordance with current guidelines (eg, American Society of Hematology 2020). This applies particularly to patients with afibrinogenemia or severe hypofibrinogenemia who have a history of recurrent thrombosis or those with type 3B dysfibrinogenemia in whom long-term full-dose anticoagulation is warranted [47]. Among the anticoagulant treatments, the utilization of direct oral anticoagulants has been increasing [17,86,[89], [90], [91], [92], [93]].

On the other hand, the use of antivitamin K medications is limited due to the complexity inherent to monitoring the International Normalized Ratio in patients with an already prolonged baseline prothrombin time, making accurate dose adjustments complex. However, some point-of-care devices, such as CoaguChek XS that utilize an electrochemical endpoint are independent of fibrinogen levels and can be used to monitor vitamin K antagonists [94]. Some authors argue that the use of direct anti-IIa drugs may offer a greater inhibition of the elevated levels of circulating thrombin in afibrinogenemia, but no specific data are yet available to support this claim [95]. We consider that patients with afibrinogenemia or severe hypofibrinogenemia receiving antithrombotic therapy face a bleeding risk comparable to that of individuals with hemophilia. Therefore, we believe that minimizing the duration of dual antiplatelet therapy, followed by long-term aspirin monotherapy, could be recommended [96,97]. However, further studies are needed to assess its efficacy and safety.

As previously mentioned, postsurgical and pregnancy-related settings confer high thrombotic risk, particularly in afibrinogenemia and severe hypofibrinogenemia [18]. Accordingly, the administration of thromboprophylaxis is imperative and should be determined on the basis of a multidisciplinary evaluation, ensuring that treatment strategies are tailored to the patient’s needs and risk factors (Table 3). In cases of afibrinogenemia and severe hypofibrinogenemia, fibrinogen replacement targeting a fibrinogen plasma level > 1 g/L is essential to cover the duration of pharmacologic thromboprophylaxis [88]. In 3A dysfibrinogenemia, mechanical thromboprophylaxis is recommended when the bleeding risk is high. For 3B dysfibrinogenemia, pharmaceutical thromboprophylaxis is mandatory and should be sustained [44].

**Table 3:** Modalities of management and prevention of thrombosis in congenital fibrinogen disorders.

Type of fibrinogen disorder	Venous thrombosis^a^	Arterial thrombosis^a^	Thromboprophylaxis^a^
Afibrinogenemia and severe hypofibrinogenemia	Limited duration, under fibrinogen supplementation targeting > 1 g/L	Avoid dual antiplatelet therapy when possible, under fibrinogen supplementation targeting > 1 g/L	Accurate pharmacologic thromboprophylaxis under fibrinogen supplementation targeting > 1 g/L
Mild to moderate hypofibrinogenemia^b^	Limited duration if possible	Shortest course of dual antiplatelet therapy possible	Mechanical thromboprophylaxis over pharmacologic
Dysfibrinogenemia 3A	Limited duration if possible	Shortest course of dual antiplatelet therapy possible	Mechanical thromboprophylaxis over pharmacologic
Dysfibrinogenemia 3B	Long-term anticoagulation	In addition to antiplatelet, long-term anticoagulation if indicated	Accurate pharmacologic thromboprophylaxis

a The general recommendation regarding modality and length for the general population should be adapted. An individual assessment of the bleeding risk based on personal and familial history is necessary.

b Moderate hypofibrinogenemia can require a fibrinogen supplementation targeting > 1 g/L.

## 7 Conclusion

Thrombotic events in patients with CFDs exhibit significant variability, with reported prevalence rates differing widely depending on the type of disorder. Thrombotic events in CFDs occur in diverse locations, from common venous sites to arterial district such as the aorta and digital arteries.

Genetic mutations, such as those associated with impaired binding of thrombin or fibrinolysis factors, and extrinsic factors such as surgery, fibrinogen replacement, and pregnancy, all contribute to the variability in thrombosis risk in CFDs. Further research is needed to clarify how these factors interact and thus to refine management strategies.

The management of thrombotic events in patients with CFDs requires an individualized approach that carefully balances bleeding risk and prothrombotic tendencies. Anticoagulation and thromboprophylaxis must be individualized, with careful consideration of patient-specific factors and the type of fibrinogen disorder.

The mechanisms driving thrombotic complications after fibrinogen replacement remain inadequately defined. Although this therapy is primarily used to prevent bleeding, it may also regulate thrombin levels in afibrinogenemia; however, this possibility remains to be fully elucidated. Additionally, the influence of specific genetic mutations and dysfibrinogenemia subtypes on long-term thrombotic risk and recurrence remains insufficiently characterized in CFDs.

Improving our understanding of the multifactorial nature of thrombosis in CFDs is essential to refine risk assessment and optimize patient management. Targeted research is needed to fill current knowledge gaps and guide evidence-based therapeutic strategies.
